# Demographic profile and susceptibility pattern of bacterial isolates from endotracheal aspirates of ventilator assisted patients admitted in the medical intensive care unit of a tertiary hospital: a retrospective cross sectional study

**DOI:** 10.1186/2047-2994-4-S1-P240

**Published:** 2015-06-16

**Authors:** PBN Gaffud, P Salandanan

**Affiliations:** 1 Internal Medicine, Cardinal Santos Medical Center, San Juan, Philippines

## Introduction

Hospital-acquired, ventilator-associated pneumonia, and healthcare-associated pneumonia are important causes of morbidity and mortality despite improved antimicrobial therapy, supportive care, and prevention.[1] Empiric antibiotic treatment is widespread especially in patients admitted in ICU, leading to multiple resistances of bacteria to certain drugs. Hence, this study aims to provide local data for bacterial isolates among ventilator assisted ICU patients, leading to a better choice of initiating antibiotic coverage by the physicians and medical residents in relation to patients’ demographic profile.

## Objectives

To describe the demographic profile and susceptibility pattern of bacterial isolates from endotracheal tube aspirates (ETA) of patients admitted in the Medical ICU of Cardinal Santos Medical Center.

## Methods

This is a retrospective cross sectional study. All adult patients who were intubated and admitted in the Medical ICU from January 2013-December 2013 were included. Data were reviewed using charts and bacterial isolates through computer generated census in the Section of Microbiology.

Frequencies and percentages were used to summarize nominal data. Means and standard deviations were computed for continuous variables. To test for statistical significance, Fisher’s exact test was used for categorical variables.

## Results

Over a 12 month period, 157 intubated patients were admitted in the Medical ICU and 59 patients met the inclusion criteria. Most common isolates identified were *Klebsiella pneumoniae* (26.47%) and *Pseudomonas aeruginosa* (25%).

## Conclusion

Hypertension, diabetes mellitus and malignancy are the common co-morbid conditions among patients admitted in the ICU with pneumonia. Susceptibility patterns of the different organisms identified in the study showed highest sensitivity with Meropenem (73.5%). Prolonged hospitalization and ICU stay increases the risk of patients for developing *Escherichia coli* and the reasonable empiric treatment options are Carbapenems. Continued antibiotic resistance surveillance should be performed.

## Disclosure of interest

None declared.
